# Fungi and the Rise of Mammals

**DOI:** 10.1371/journal.ppat.1002808

**Published:** 2012-08-16

**Authors:** Arturo Casadevall

**Affiliations:** Departments of Microbiology and Immunology and Medicine, Division of Infectious Diseases, Albert Einstein College of Medicine, Bronx, New York, United States of America; Duke University Medical Center, United States of America

## The Mammalian Lifestyle Is Energetically Costly

Here are two indisputable facts: we are living in the age of mammals [1], and immunologically intact mammals are highly resistant to fungal diseases, such that most human systemic fungal are considered “opportunistic” [2]. Could these two facts be connected? The mammalian lifestyle is characterized by endothermy, homeothermy, and care for the young, including nourishment via lactation, all of which are energetically costly activities. In contrast, reptiles, which are ectotherms, require about one-tenth of the daily mammalian energy needs [3], and reptilian development is faster and requires less parental involvement. Given this energy handicap, how did mammals replace reptiles as the dominant land animals? This essay further develops the hypothesis originally proposed seven years ago that fungi contributed to the emergence of mammals by creating a fungal filter at the end of the Cretaceous that selected for the mammalian lifestyle and against reptiles [4].

## Mammals Are Naturally Resistant to Fungal Diseases

Mammals are highly resistant to systemic fungal diseases. Whereas dermatophyte-associated diseases are common, these are seldom life threatening. For humans most fungal diseases were described in the 20^th^ century and are associated with changes in the host such as iatrogenic immunosuppression, antibiotic-mediated disruption of the microflora, or other immune-impairing conditions as HIV infection, hematologic malignancies, and rheumatologic conditions. Unlike viral and bacterial diseases human mycoses are seldom contagious.

Endothermy and homeothermy are thought to contribute to mammalian resistance to mycosis by creating a thermal exclusionary zone that inhibits most fungal species [5]. The remarkable resistance of mammals to mycotic diseases is probably a combination of a vertebrate immune system, with both innate and adaptive arms, and elevated body temperatures. Experimental evidence for the synergy of temperature and immunity is apparent from studies of cryptococcal infection in rabbits, which have core temperatures of 40–41°C [6]. Rabbits are naturally resistant to systemic *C. neoformans* infection. However, rabbits can be infected with *C. neoformans* when inoculated in the skin or cornea, which are cooler, but the fungus does not disseminate. However, when rabbits are immunosuppressed with corticosteroids, *C. neoformans* infection is rapidly fatal [7].

Primitive mammals like the platypus, with core temperatures near 32°C, are susceptible to *Mucor amphibiorum*, a fungus with a maximal thermal tolerance of 36°C that would make it avirulent for higher mammals [8]. The resistance of mammals to fungal diseases is in sharp contrast to the vulnerability of other vertebrates, such as amphibians, a group that is currently under severe pressure from a chrytrid [9]. Like mammals, amphibians have adaptive immunity, but unlike mammals, they are ectotherms and lack a thermal environment that is exclusionary to fungi. Hence, their vulnerability to fungal diseases echoes the experimental findings in rabbits whereby high resistance is conferred by a combination of high temperature and vertebrate-level immunity [6]. Amphibians can be cured of chrytridomycosis if placed at 37°C [10]. Another example of the protection provided by the combination of vertebrate-level immunity and endothermy comes from bats. In the summer bats manifest high activity and mammalian temperatures, but during winter hibernation their core temperatures drop as they hibernate and become vulnerable to infection with *Geomyces destructans*, a fungus that is decimating several North American bat species [11]. Infected bats woken from hibernation made full recovery when provided with supportive care, as higher body temperature inhibited fungal growth [12]. It is noteworthy that birds, which are also endotherms, are susceptible to *Aspergillus fumigatus* [13], a thermotolerant fungus that can survive up to 55°C [14].

A computation of the optimal temperature that would provide maximal protection against fungi given the caloric needs needed to maintain elevated temperatures yielded a value of 36.7°C, which is very close to mammalian temperatures [13]. This raises the possibility that mammalian resistance to fungi through the combination of vertebrate-level immunity and endothermy could have been the result of selection by pathogenic fungi.

## A Fungal Filter at the Cretaceous-Tertiary (K-T) Boundary

Mammals replaced reptiles as the dominant land forms after the catastrophe that marked the end of the Cretaceous and the beginning of the Tertiary, an event known as the K-T boundary. The currently favored hypothesis for the demise of dinosaurs and end of the age of reptiles is a bolide impact approximately 65 million year ago with the possibility that other events, such as increased volcanism, contributed to disrupting the cretaceous ecosystem [15]. That ecological calamity was accompanied by massive deforestation [16], an event followed by a fungal bloom [17], as the earth became a massive compost. Although one cannot know which spores were present at the time, the likelihood that pathogenic fungi existed at the K-T boundary is enhanced by the finding that the potential for pathogenicity probably arose independently several times in evolution [18].

There is now increasing evidence that large dinosaurs were warm bodied [19], as a result of their size, which would have entailed considerable heat generation dependent on food metabolism and their metabolic activities. Large animals at the top of the food chain, such as dinosaurs, are highly vulnerable to ecosystem disruption. The altered ecosystem would have implied disruption of food sources and changed climate, which is thought to have included a significant cooling of the earth [20], by dust clouds and fires. Such stresses would be expected to weaken any survivors of the bolide blast with consequent immunological impairment and could have made survivors, and their eggs, susceptible to fungal diseases, especially if they could not maintain body warmth in the setting of starvation.

Since there are reptiles today, it is clear that some reptiles survived the K-T boundary cataclysm. This raises the question, if reptiles were previously so successful, why did they not reclaim the earth to launch a second reptilian age? It is difficult to imagine how mammals could have replaced reptiles as the dominant land forms without some selection mechanism for this energetically costly lifestyle. This led me to propose the hypothesis that fungal proliferation after the devastation of the KT event preferentially selected for the fungal-resistant endothermic and hindered the re-emergence of a second reptilian age [4]. Although we do not know the timeline for the recovery of the planet climate, it is estimated that photosynthesis was shut down for 6 months and climate cooling persisted for at least 9 years [20], and the occurrence of a fungal bloom sufficient to have left fossil evidence implies that surviving animals were exposed to massive numbers of fungal spores. The darkened skies and cooler temperatures that accompanied the K-T cataclysm [20] would have shielded the sun and reduced the ability of ectothermic creatures such as reptiles to induce fevers by insolation, a necessary activity for protection against fungal diseases. Hence, it is reasonable to posit that ectothermic creatures unable to induce behavioral fevers and in weakened states from environmental stress would have been at a severe disadvantage relative to small mammals with their innate thermal exclusionary zones for fungal growth. Further complicating the situation for reptiles is that eggs can be vulnerable to fungal attack [21], whereas mammalian progeny would be protected in placentae.

## Climate Change and Future Fungal Threats

Climate warming implies that the temperate gradient from mammals and average environmental temperatures will be reduced. Higher global temperatures could select for more thermally tolerant fungi, and it is possible that many fungi with current pathogenic potential for mammals that are unable to cause disease in mammals due to thermal intolerance will acquire the capacity to survive at mammalian temperatures and thus become pathogenic for mammals [22]. This concern is heightened by the fact that some fungi can be easily adapted to higher temperatures by thermal selection, as exemplified by the generation of a thermally resistant entopathogenic fungus as an attempt to create a pest control strain that would be less susceptible to insect-induced behavioral fevers [23].

## The Fungal-Mammalian Emergence Hypothesis in Context

The fungal-mammalian emergence hypothesis posits that fungi selected for the emergence of mammals. The hypothesis suggests an explanation for how the highly energy-intensive mammalian lifestyle was selected and for the relative resistance of immunologically intact mammals to fungal diseases. The hypothesis is a plausible synthesis assembled from very disparate lines of evidence. At this time it is unlikely that experimental evidence will be available in the near future to validate or refute this hypothesis simply by the very nature of what it tries to explain, and the remoteness of past events. For example, given that the animals that died as a result of the KT-related events represent an extremely small part of the fossil record, it is unrealistic to imagine finding fossils that could unequivocally be dated to the time in question with evidence of fungal disease. Fungal diseases can leave traces in the fossil record, as manifested by the finding of *Coccidioides*-like spherules in a fossil bison from the Holocene [24], but those fossils are very recent relative to the KT event and fungal effects on bone tissue usually reflect chronic infections. In contrast, fungal diseases caused by microscopic organisms that killed hosts by destroying soft tissues would leave no fossil record. On the other hand, recent developments with amphibian chrytridmycosis and the white nose syndrome in bats provide strong circumstantial evidence for the notion that fungal diseases could have provided strong selection pressures and driven some species to extinction. Although these are examples of individual fungal-host interaction in specialized ecological settings, they do provide precedents for the notion that fungi can be powerful selective forces for vertebrate species. In addition, there is now considerable evidence that fungi are potential threats to entire ecosystems [25]. The fungal-mammalian emergence hypothesis will likely continue to evolve as new information is available and is best considered as a cognitive tool for stimulating thinking and discussion on global issues related to evolutionary selection, infectious diseases, and ecological change.
